# PHYSIOLOGICAL DYSREGULATION AS A PREDICTOR OF COGNITIVE DECLINE

**DOI:** 10.1093/geroni/igac059.2257

**Published:** 2022-12-20

**Authors:** Elizabeth Mahon, Sharon Stein Merkin, Margie Lachman, Arun Karlamangla, Teresa Seeman

**Affiliations:** Brandeis University, Waltham, Massachusetts, United States; University of California Los Angeles, Los Angeles, California, United States; Brandeis University, Waltham, Massachusetts, United States; University of California, Los Angeles, Los Angeles, California, United States; University of California Los Angeles, Los Angeles, California, United States

## Abstract

Preclinical indicators of disease such as inflammation, cortisol and glucose dysregulation, and multisystem dysregulation (allostatic load) are related to individual differences in the level of cognitive functioning across adulthood. This study examined whether individual biological systems and allostatic load are related to differential patterns of change in cognitive functioning over 9 years. Data are from the Midlife in the United States (MIDUS) study second (biomarker and cognitive) and third waves (cognitive). The sample includes 863 men and women who ranged in age from 35 to 85 when the data were first collected. MIDUS biomarkers include a comprehensive range of biological and anthropometric measurements reflecting cardiovascular functioning, glucose metabolism, lipid metabolism, inflammation, HPA axis function, as well as sympathetic and parasympathetic nervous system function. Summary indices of dysregulation in each of these major systems as well as an overall index of multi-system dysregulation, or allostatic load were examined in relation to 9-year changes in episodic memory and executive functioning from the Brief Test of Adult Cognition by Telephone. Regression analyses, controlling for preexisting diseases and medications, showed that higher allostatic load was associated with decreased executive functioning over time for those who started out with higher cognitive performance at baseline, after adjusting for age, gender, race, English language, education, neurological conditions, medication use and smoking. Identifying biomarkers as antecedents of cognitive changes in midlife and old age, can potentially aid in the early detection of cognitive impairments and increase the possibilities for preventive interventions.

